# The Hospital Library and Charities Bureau

**Published:** 1904-04-09

**Authors:** 


### THE HOSPITAL LIBRARY AND CHARITIES BUREAU.

Inquiries of the librarian are answered in this column free of charge. Inquiries to be answered promptly by post must be accompanied by a fee of 2s. 6d.

The Librarian announces the addition of 24 volumes of the Encyclopædia to the Library for the use of members.

*Practical Points. Cost of Out-patients.*

If the cost of 1,000 out-patients equals that of one constantly-occupied bed, what is meant by an out-patient? Does it mean 1,000 attendances of patients, or does it mean 1,000 individuals who may attend several thousand times?

Attendances are not out-patients, and never can be. The only way to arrive at the cost of the out-patient department is to keep all the expenditure properly chargeable to the out-patient department distinct in the books from all other expenditure. Then, by dividing the whole number of out-patients into the total expenditure, you arrive at the actual cost of each out-patient.

